# Epidemiology of Pediatric Chronic Pain: An Overview of Systematic Reviews

**DOI:** 10.1007/s11916-025-01380-5

**Published:** 2025-04-01

**Authors:** Alessio Lo Cascio, Miriam Cascino, Marcella Dabbene, Antonella Paladini, Omar Viswanath, Giustino Varrassi, Roberto Latina

**Affiliations:** 1 Department of Biomedicine and Prevention, Tor Vergata University of Roma, Via Montpellier, Roma, 1 - 00133 Italy; 2 Department of Health Promotion, Mother and Child Care, Internal Medicine and Medical Specialities, University of Palermo, Palermo, 90127 Italy; 3 Department of Nursing Research and Management, La Maddalena Cancer Center, Via San Lorenzo, 312/D, Palermo, PA 90146 Italy; 4 Department of MESVA, University of L’Aquila, L’Aquila, 67100 Italy; 5 Creighton University School of Medicine, Mountain View Headache and Spine Institute, Phoenix, AZ USA; 6 Fondazione Paolo Procacci, Roma, 00193 Italy

**Keywords:** Pediatric, Chronic non-cancer pain, Epidemiology, Systematic review, Pain, Umbrella review

## Abstract

**Purpose of Review:**

Chronic non-cancer pain in children and adolescents represents a significant public health issue, affecting physical, psychological, and social well-being. Defined as pain persisting for over three months, this condition is influenced by developmental, socioeconomic, and cultural factors. However, its prevalence remains uncertain and debated. A comprehensive literature search was conducted across electronic databases, including Medline, Embase, CINAHL, PsycINFO, and the Cochrane Library. Eligible systematic reviews were critically appraised using the AMSTAR-2 tool to assess methodological quality. This overview synthesises evidence from existing systematic reviews to provide an updated understanding of the epidemiology and burden of paediatric non-cancer chronic pain.

**Recent Findings:**

Findings revealed substantial variability in the reported prevalence of specific pain types: headaches (4–83%), abdominal pain (4–53%), musculoskeletal pain (4–40%), and back/low-back pain (14–24%). Prevalence was generally lower in low- and middle-income countries, likely due to barriers in healthcare access. Methodological heterogeneity was observed across studies, and AMSTAR-2 assessment identified critical limitations in some systematic reviews, impacting the reliability of findings.

**Summary:**

This overview highlights the urgent need for standardised research methodologies to accurately monitor the prevalence of paediatric non-cancer chronic pain. Standardisation is essential for informing policies aimed at mitigating the long-term impact of chronic pain in children and adolescents. Addressing these issues, particularly in resource-limited settings, is crucial for improving health outcomes and reducing societal and economic burdens.

**Supplementary Information:**

The online version contains supplementary material available at 10.1007/s11916-025-01380-5.

## Introduction

Acute pain is a universal human experience, serving as a vital protective mechanism that alerts to potential injury or harm [1] and does not spare the pediatric population. However, when pain extends beyond its acute phase, it can devolve into a chronic condition, with far-reaching impacts for individuals and society at large. Chronic pain, defined as pain persisting or recurring for more than three months, significantly affects quality of life, functional capacity, and socioeconomic status across both adult and pediatric populations [2].

Pediatric non-cancer chronic pain encompasses a broad spectrum of conditions, including, but not limited to, headaches, abdominal pain, and musculoskeletal pain, often featuring overlapping symptoms and multi-factorial etiologies [3]. The complexity is further exacerbated by the unique developmental stages of childhood and adolescence, where pain experiences can profoundly influence physical, emotional, and social development [4, 5]. This condition poses a multifaceted health challenge, adversely affecting children’s quality of life, functional abilities, and economic well-being, thereby creating extensive burdens on patients and their communities.

Chronic pain impacts children beyond physical discomfort. Education activities often suffers due to increased school absenteeism and reduced concentration [6, 7]. The implications during these critical formative years extend beyond immediate suffering; chronic pain in childhood is a strong predictor of chronic pain in adulthood, potentially perpetuating a cycle of ongoing disability and reduced quality of life [8, 9]. Moreover, the impact on families is profound, as parents and siblings often face increased stress, financial hardship, and disruptions to family dynamics [10, 11]. From a public health perspective, pediatric chronic pain constitutes a significant issue with long-term ramifications for health systems and society. The economic burden associated with pediatric chronic pain is considerable, encompassing direct healthcare costs, loss of productivity among caregivers, and potential long-term impacts on affected individuals [12, 13]. Despite the magnitude of this problem, there remains a notable disparity in resource allocation and research attention toward pediatric chronic pain compared to other pediatric health conditions [14]. Globally, the landscape of pediatric chronic pain remains incomplete, particularly in low- and middle-income countries (LMICs), where resources for pain management are often scarce [4]. In those regions, multidisciplinary approaches to pain treatment are rarely available, and even basic analgesic medications may be limited [15]. The global nature of this problem necessitates a comprehensive understanding of the epidemiology of pediatric chronic pain across diverse socioeconomic contexts to inform targeted interventions and policy decisions [16]. Despite the pressing need, there are few literature reviews focusing on the prevalence of pediatric chronic pain. Existing data lack clarity and consistency, with variations across different age groups, regions, and socioeconomic strata. Addressing these challenges and filling existing knowledge gaps requires the collection and critical evaluation of available literature through systematic reviews and meta-analyses. By identifying inconsistencies and harmonizing data, we can develop a clearer understanding of the impact of pediatric chronic pain. This approach not only facilitates the formulation of evidence-based policies but also highlights areas requiring further research and intervention. The objective of this overview is to provide an updated review of systematic reviews describing the epidemiology of pediatric chronic pain.

## Materials and Methods

### Design and Search Strategy

**Fig. 1:**
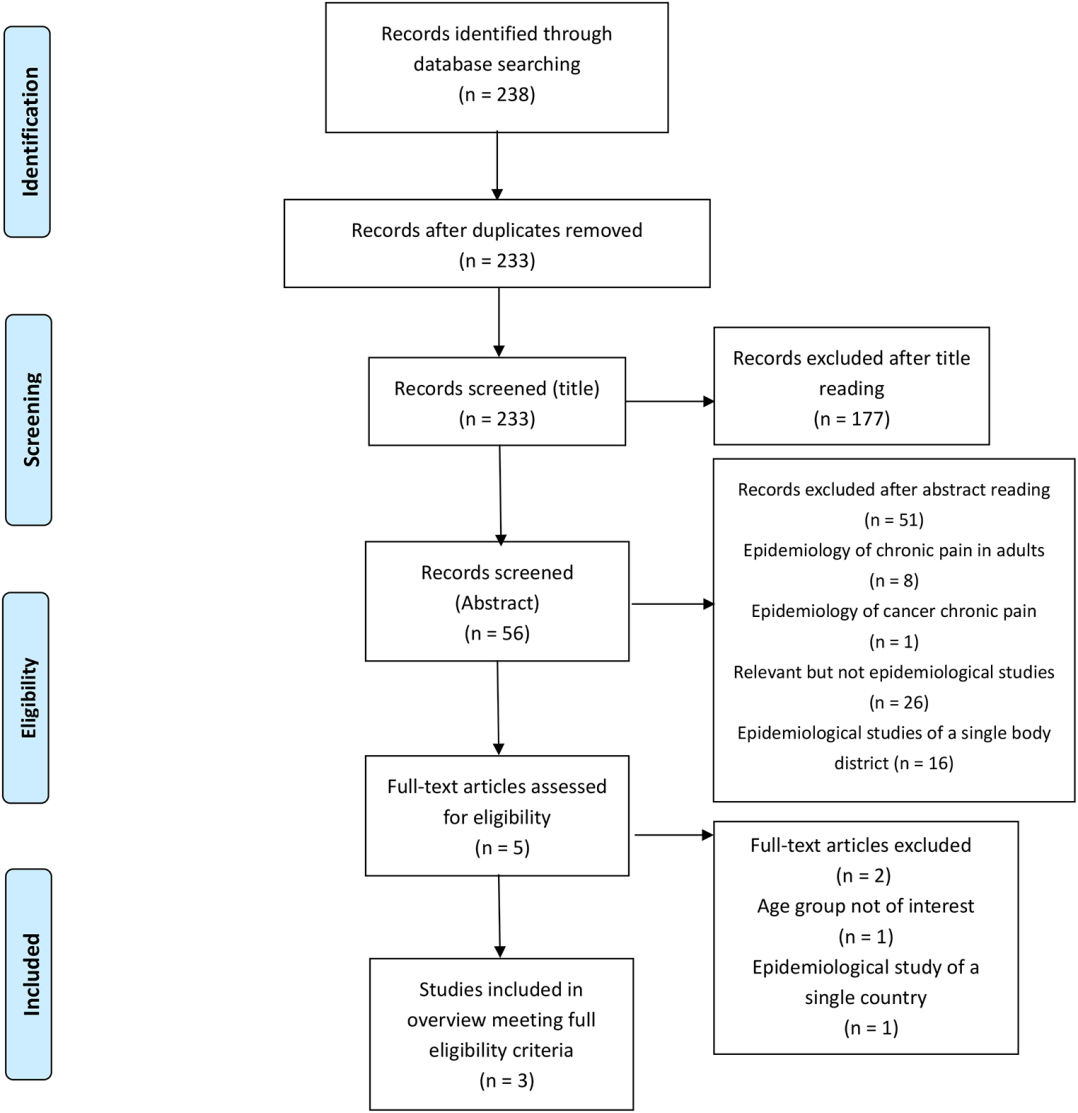
Prisma. From: Moher D, Liberati A, Tetzlaff J, Altman DG, The PRISMA Group (2009). Preferred Reporting Items for Systematic Reviews and Meta-Analyses: The PRISMA Statement. PLoS Med 6(6): e1000097. doi:10.1371/journal.pmed1000097. For more information, visit www.prisma-statement.org

**Fig. 2:**
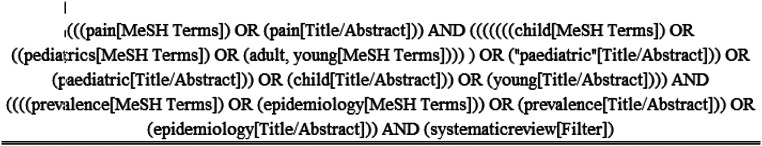
Search strategy

This overview of a systematic review (SR) of the literature was conducted adhering to the guidelines set forth by Preferred Reporting Items for Systematic Reviews and Meta-analyses (PRISMA) statement [17, 18] (Fig. 1). To ensure a thorough and methodical approach, an advanced search strategy was devised and implemented across a range of electronic databases. These included, but were not limited to, Medline, Embase, Science Citation Index, CINAHL, and the Cochrane Library. The search methodology employed a carefully curated selection of keywords, terms, and Medical Subject Headings (MeSH), which were systematically combined using the Boolean operators ‘AND’ and ‘OR’ (Fig. 2). Search terms were formatted as necessary for each database. This intricate search framework facilitated the identification and retrieval of pertinent records, thus ensuring a comprehensive and robust literature review.

### Systematic Review Protocol Registration

In line with international recommendations and to ensure the highest levels of transparency and integrity, the protocol for this overview of systematic reviews was registered with the International Prospective Register of Systematic Reviews (PROSPERO) database (https://www.crd.york.ac.uk/prospero/) managed by the National Institute for Health Research under protocol number CRD42024608716 prior to commencing the literature search [19].

### Eligibility Criteria

Systematic reviews that aligned with the primary objective of describing the epidemiology of pediatric chronic pain were considered. Specifically, systematic reviews addressing (1) chronic pain in various pediatric body regions, and (2) non-cancer pediatric chronic pain, were evaluated. Only reviews published in English or Italian were included, and the selected studies had to be either systematic reviews or meta-analyses. No restrictions were placed on the publication date to ensure a comprehensive collection of information.

### Data Extraction

A total of 238 systematic reviews were identified. Two independent researchers (ALC and MC) screened titles and abstracts for relevance. Discrepancies were resolved through discussion, and full texts of selected publications were retrieved for further analysis.

### Quality Appraisal of Reviews

The AMSTAR-2 tool was employed to assess the methodological quality of the included reviews [20]. This tool evaluates 16 items, with seven considered critical, and provides quality ratings of high, moderate, low, or critically low. AMSTAR-2 ensures a rigorous evaluation, identifying methodological flaws and supporting robust data interpretation [20].

## Results

Of the 238 articles identified, 5 duplicates were removed. Among the remaining 233, 177 systematic reviews were excluded based on title relevance to the topic of interest. Of the 56 that remained, 51 were excluded after abstract review: 8 addressed the epidemiology of chronic pain in the adult population, 26 were pertinent but not strictly epidemiological studies, 16 focused on non-cancer chronic pain epidemiology in specific body district, and 1 addressed pediatric chronic oncological pain. Of the final five systematic reviews, one was excluded as it described chronic pain epidemiology in adolescents and young adults aged 15 to 34 years, and another was excluded as it described chronic pain epidemiology of a single country. Upon initial evaluation, it was impossible to disaggregate data to ascertain chronic pain rates specific to the study’s population (Appendix 1). Ultimately, 3 relevant reviews were included: one described the epidemiology of pediatric non-cancer chronic pain in different body regions, while the other focused on pediatric non-cancer chronic pain in low- and middle-income countries, as outlined in Table 1.

### Definition of Chronic Non Cancer Pain

The included reviews employed varying definitions of chronic non-cancer pain. For instance, King et al. [21] considered pain persisting for periods ranging from one week to six months, while Liao et al. [22] and Chambers et al. [23] focused on pain lasting at least three months.

### Prevalence of Pediatric Non-Cancer Chronic Pain

The prevalence of non-cancer chronic pain in pediatric populations was examined in three reviews. King et al. [21] did not report an overall prevalence in the reference population but includes different type of chronic pain prevalence in different district of body. In contrast, Liao et al. [22] reported a pooled mean prevalence of chronic pain of 8% (95% CI: 6–10%) across the included studies, with prevalence rates ranging from 0 to 36% in boys and from 0 to 53% in girls. This result refers in a low- and middle-income countries. Chambers et al. [23] identified a prevalence of 20.8% (95% CI: 19.2–22.4%) and observed that girls have a higher prevalence of chronic pain (18.3%) compared to boys (12.7%).

### Quality of Studies

Using AMSTAR-2, we assessed our confidence in the included reviews as “high,” “critically low,” and “low,” respectively. The systematic review by King et al. [21] did not satisfy three critical items and one non-critical item. Regarding the systematic review by Chambers et al. [23], we found that only one critical item was not satisfied, as shown in Table 2.

### Settings of Included Studies

The three systematic reviews examined present diverse configurations in the contexts of the included studies King et al. [21] primarily included studies from high-income countries such as the United States, Finland, Germany, Sweden, the United Kingdom, and the Netherlands, while also incorporating a few middle-income countries like Brazil, Turkey, and Malaysia. Liao et al. [22] offers a broader geographical coverage, encompassing high-, middle-, and low-income countries including Iran, Nigeria, Tunisia, India, and China. Similarly, Chambers et al. [23] included a wide range of countries, covering numerous low- and middle-income nations such as Nigeria, Tanzania, and Bangladesh, alongside high-income countries.

### Prevalence of Headache

The overall prevalence of headache reported by King et al. [21] ranged from 8 to 83%, identifying a correlation between lower socioeconomic status and higher prevalence rates of pain, particularly headaches. This finding appears different compared to the average prevalence rate of headaches reported by Liao et al. [22], which was 4%. In the systematic review by Chambers et al. [23], the prevalence of headache varied from 0.7 to 70%.

### Prevalence of Abdominal Pain

King et al. [21] found that abdominal pain prevalence ranged from 4 to 53%. Liao et al. [22], reported a prevalence rate of 7%, while the systematic review by Chambers et al. [23], found a prevalence of 17.3%.

### Prevalence of Musculoskeletal Pain and Back Pain

According to the systematic review by King et al. [21], the prevalence of back pain ranged from 14 to 24%, while musculoskeletal pain ranged from 4 to 40%. In low- and middle-income countries, Liao et al. [22], identified a prevalence of musculoskeletal pain at 9%. Chambers et al. [23] reported an overall prevalence of back pain at 19.1% and musculoskeletal pain at approximately 25.7%.

### Prevalence in Range of Age

This overview revealed that the pediatric population studied had an age range of 0–19 years, with the majority of studies focusing on children aged 5 years and older. Chronic pain is less common in young population (0–4 years), whereas adolescents are more frequently affected. Chronic pain is often associated with older patients, but 25% of pediatric patients are estimated to experience chronic pain. The age group most commonly affected by headaches was 7 years and older, while abdominal pain was more prevalent in children older than 8 years.

### Risk of Bias Assessment

Among the three reviews, only two [22, 23] assessed risk of bias by performing meta-analyses of prevalence data. Chambers et al. [23] included studies classified as having low risk of bias (47.9%), moderate risk (46.2%), and high risk (5.9%). The most common biases were lack of national representation and nonresponse bias. Liao et al. [22], found that over 95% of included studies had a low overall risk of bias, while only one study had moderate risk.

**Table 1:** Descriptive summary of systematic reviews included

Author and Year	Databases	Number and type of Included Studies/	Publication Period of Included Studies	Settings of Included Studies	Data Collection Methods in Included Studies	Definition of Chronicnon cancer Pain	Total Population	Meta-analysis	Age Range (years)
King et al., 2011	EMBASE, Medline, CINAHL, and PsycINFO	32 studies: 23 cross-sectional, 7 prospective, 2 retrospective	From 1993 to 2008	United States, Brazil, Finland, Germany, Turkey, Sweden, Netherlands, Iceland, Malaysia, Singapore, United Kingdom, Greece, Spain	9/32 interviews; 23/32 questionnaires	Pain persisting for 6 months; ≥3 months; within the last year; within the last month; within the last week; point pain	94,357	No	0–19
Liao et al., 2022	Medline, Embase, CINAHL, PsycINFO, Web of Science, Cochrane, WHO Global Index Medicus	27 studies: 24 cross-sectional, 2 prospective, 1 retrospective	From 1985 to 2020	Iran, Brazil, Nigeria, Tunisia, Malaysia, Turkey, Zimbabwe, Ecuador, Sri Lanka, Armenia, Albania, TFYR Macedonia, Bulgaria, Moldova, Russia, Ukraine, India, China, Thailand, Tanzania	7/27 interviews; 20/27 questionnaires	Pain persisting ≥ 3 months; 6 months or more; last year	82,016	Yes	0–19
Chambers et al., 2024	EMBASE, PubMed, CINAHL, and PsycINFO	119 studies: 104 cross-sectional, 12 longitudinal, 3 repeated	From 2009 to 2023	Nigeria, Brazil, Kuwait, Saudi Arabia, Turkey, Jordan, Australia, Portugal, India, Greece, USA, Italy, Zimbabwe, Singapore, Croatia, Sri Lanka, Mexico, Denmark, New Zealand, Israel, Finland, Norway, Russia, Ecuador, Indonesia, Japan, Cameroon, Serbia, Panama, Belgium, Spain, South Korea, Bangladesh, Colombia, China, Iran, Thailand, Hong Kong, Sweden, Netherlands, El Salvador, Curaçao	6/119interviews; 113/119questionnaires	Pain with a minimum duration of at least 3 months; pain described as chronic, persistent, or recurrent without reported time interval	1,043,878	Yes	0–19

## Discussion

This overview describes the prevalence of pediatric non-cancer chronic pain across different regions, socioeconomic contexts, and among girls [22, 23].

The primary objective of this overview was to synthesize the epidemiology of pediatric non-cancer chronic pain from 178 primary studies included in three systematic reviews. Understanding these prevalence rates is crucial for public health planning and for tailoring healthcare policies in different countries to provide specialized services for pain management [24]. Furthermore, reporting the clinical characteristics of pediatric patients is vital as it offers valuable feedback to pain management professionals, especially those in pain clinics within a structured network. Such data can promote advanced nursing practices and the development of specialized, globally-informed care strategies pediatric [25].

The three systematic reviews included in this overview cover studies over a broad time span from 1993 to 2023. Over the years, there has been an evolution in the international understanding and definition of chronic pain, which is reflected in the temporal spread of the included reviews. King et al. [21] employed the definition of chronic pain, as described by IASP [26]. In contrast, the more recent reviews by Liao et al. [22] and Chambers et al. [23] reflect a shift towards greater standardization, aligning with the International Classification of Diseases, 11th Revision (ICD-11), which defines chronic pain as pain persisting or recurring for at least three months [2]. Their inclusion criteria allowed for varied definitions of chronic pain, with pain duration ranging from one month to six months, or even pain experienced within the last week or year. This lack of standardization likely contributed to the wide range of prevalence rates reported in their reviews. This standardization is crucial for improving the comparability of studies and for accurately assessing the prevalence, the methodologies and interpretations of data. Our results highlight that the revision of King et al. [21] did not provide an overall prevalence rate, in contrast Liao et al. [22] reported a pooled mean prevalence of 8%, and Chambers et al. [23] reported an overall prevalence of 20.8%.

Evidence suggests that lower socioeconomic development and, in some cases, sociocultural factors, contribute to the rising incidence of chronic pain [27], contrary to findings in one of the included reviews [22]. In low- and middle-income countries (LMICs), challenges such as resource constraints, limited access to specialized care, and inadequate healthcare infrastructure are compounded by cultural beliefs and stigma that may discourage pain reporting or care-seeking behavior [28].

The public health implications are particularly concerning in LMICs. Undiagnosed or untreated chronic pain interferes with education, reduces academic achievement, and limits future socioeconomic opportunities. Additionally, it places emotional and financial stress on families, especially in resource-scarce settings [9].

Addressing chronic pain in pediatric populations is critical to improving individual health outcomes and mitigating broader societal impacts [29]. In this overview it emerges that the pediatric population studied has an age range that goes from 0 to 19 years. However, it emerges that the age phase of the pediatric population most represented has a range that is greater than 5 years. It is likely that chronic pain in such a young population (0–4 years) is low and that the adolescent population may be affected by it. Chronic pain is normally attributed and identified only to older patients. It is estimated that about 25% of pediatric patients suffer from chronic pain [30], and these findings confirm that, differently than expected, it is a common complaint in childhood and adolescence, the most affected age group represented by adolescents between 12 and 15 years of age [21]. The review also identified several gaps in the understanding of the epidemiology of chronic pain in children and adolescents, including restricted age ranges and lack of longitudinal studies. The first step to take in order to avoid any possible underestimation of the importance of properly treating pediatric pain is to use necessary and reliable tools in different clinical settings [30].

Unrealistic expectations regarding treatment outcomes can lead to dissatisfaction, emphasizing the need for healthcare professionals to set clear expectations as part of pain management strategies [29]. Managing family expectations regarding treatments and ensuring effective communication by health professionals are important for effective treatment and for supporting families’ psychosocial needs it results in a trusting relationship. Health and social care systems for children and young people’s chronic pain require modification to ensure that a more holistic approach to addressing pain in children and young people is adopted, which considers all biopsychosocial, family-centered health, and social care systems [12, 31].

In the systematic reviews by King et al. [21] and Liao et al. [22] the prevalence of headache, abdominal pain, and multi-site pain was higher in girls compared with boys, and no gender differences were observed in musculoskeletal pain [21]. In contrast, Chambers et al. [23] reported that the prevalence of all types of chronic pain localizationis more prevalent in females than in males. Differences between men and women regarding pain involve anatomical, physiological, neural, hormonal, psychological, social, and cultural factors. When examining those factors, it is found that women report pain more frequently and have a lower threshold for pain than men. They usually experience more muscle-skeletal, neuropathic, electrical shock, and temperature-related pain but respond better to opioids [32]. In terms of sex differences, prevalence of headache, abdominal pain, and multi-site pain was higher in girls compared with boys.

The quality of the included systematic reviews was assessed using the AMSTAR-2 tool. King et al. [21] was rated as having “critically low” methodological quality, as it did not satisfy three critical domains and one non-critical domain. This may limit the reliability of its findings and highlights the importance of rigorous methodological standards in systematic reviews. Chambers et al. [23] was rated as “low” quality, missing only one critical domain. Liao et al. [22] was assessed as “high” quality, indicating strong methodological rigor and increasing confidence in its findings. As shown in Table 2, the three reviews have different reporting qualities using AMSTAR-2. It is possible that the methodological rigor is more evident in the reviews published from 2016 onwards, or that the methodological rigor in place was not explicitly described. A systematic review needs to use a transparently formulated query that uses systematic and rigorous methods to recognize, collect and select, and critically appraise relevant research, and to analyze information from each of the studies that are enclosed [33]. Moreover, only two reviews [22, 23] describe their risk of bias, as their meta-analysis. The most common areas of bias across both studies were lack of national representation and non-response bias [22, 23]. Risk of bias assessment is essential to establish transparency of evidence synthesis results and findings and is a defining element of systematic reviews, often performed for each included study in the review [34].

**Table 2:** Items of AMSTAR 2

Authors	1	2*	3	4*	5	6	7	8	9*	10	11*	12	13*	14**	15*	16	Rating Quality
King et al., (2011)	Y	N	Y	Y	Y	Y	N	Y	N	N	NA	NA	N	Y	NA	Y	Critically Low
Liao et al., (2022)	Y	Y	N	Y	Y	Y	Y	Y	PY	PY	Y	Y	Y	Y	Y	Y	High
Chembers et al., (2024)	Y	Y	Y	PY	Y	Y	N	Y	Y	Y	Y	Y	Y	Y	Y	Y	Low

### Limits

Several limitations are inherent in this overview. The heterogeneity of the included studies, in terms of study design, definitions of chronic pain, makes direct comparisons challenging. The evolution of the definition of chronic pain over time further complicates comparisons between older and newer studies. Additionally, cultural differences in pain perception and reporting can influence prevalence rates. As a result, chronic pain in many countries is likely underestimated due to sociocultural factors of each individual nation, potentially biasing the prevalence data. In LMICs, under-diagnosis and underreporting are likely due to limited access to healthcare services and differing cultural attitudes toward pain and illness. Another limitation found is that of not being able to describe the impact that pediatric chronic pain has on parents and family members, aspects that can be addressed through qualitative clinical research techniques. Another limitation is the potential for publication bias. Studies reporting higher prevalence rates may be more likely to be published than those with lower rates, skewing the overall prevalence estimates. The quality assessment also revealed that not all systematic reviews met the highest standards of methodological rigor, which may affect the reliability of their findings.

## Conclusions

This overview underscores the significant burden of chronic pain in the pediatric population worldwide and highlights the urgent need for public health interventions. Standardizing definitions and methodologies in chronic pain research is essential for accurate prevalence estimates and effective management strategies. Addressing chronic pain, particularly in LMICs, is critical to improving health outcomes and reducing societal and economic burdens. Future research should prioritize standardized methodologies, culturally sensitive tools, and interventions tailored to the needs of diverse pediatric populations.

## Electronic Supplementary Material

Below is the link to the electronic supplementary material.

Supplementary Material 1

## Data Availability

Data is provided within the manuscript or supplementary information files.

## Declarations

### Use of AI Tools

The authors declare they have not used Artificial Intelligence (AI) tools in the creation of this article.
