# Supplementary material for: Epidemiology of Pediatric Chronic Pain: An Overview of Systematic Reviews
Source: Curr Pain Headache Rep. 2025 Apr 1;29(1):71. doi: 10.1007/s11916-025-01380-5 (PMC11961504; doi:10.1007/s11916-025-01380-5)
Supplement: Supplementary file 1 — Supplementary Material 1 [file 11916_2025_1380_MOESM1_ESM.pdf]

Appendix 1. List of studies excluded at full-text screening stage.

Study	Pubmed ID or DOI	Reason for Exclusion
Ruzbarsky et al(2016)	*26709689*	*excluded based on title relevance to the topic of interest*
Mitsuhashi et al (2022)	*36612891*	*excluded based on title relevance to the topic of interest*
Onofri et al (2023)	*36782182*	*Records excluded after abstract reading*
Taddio et al (2022)	*36283899*	*excluded based on title relevance to the topic of interest*
Fayaz et al (2016)	*27324708*	*Epidemiological study of a single country*
DelRosso et al (2021)	*33341437*	*excluded based on title relevance to the topic of interest*
Evans et al (2022)	*35080267*	*excluded based on title relevance to the topic of interest*
Pellegrino et al (2022)	*36107254*	*excluded based on title relevance to the topic of interest*
Zheng et al (2018)	*29850535*	*excluded based on title relevance to the topic of interest*
Stiglic and Viner (2019)	*30606703*	*excluded based on title relevance to the topic of interest*
Venekamp et al (2023)	*37965923*	*excluded based on title relevance to the topic of interest*
Lopez-Leon et al (2021)	*34373540*	*excluded based on title relevance to the topic of interest*
Burch et al (2021)	*33349955*	
Lotan et al (20119)	*30691751*	*excluded based on title relevance to the topic of interest*
Shah et al (2017)	*28763554*	*excluded based on title relevance to the topic of interest*
Stoicea et al (2019)	*31096439*	*excluded based on title relevance to the topic of interest*

Chiaffarino et al (2021)	*32949284*	*non-cancer chronic pain epidemiology in specific body district,*
Spinks et al (2021)	*34881426*	*excluded based on title relevance to the topic of interest*
Feijen et al (2020)	*31935141*	*excluded based on title relevance to the topic of interest*
Yeomans et al (2018)	*29299876*	*excluded based on title relevance to the topic of interest*
Zeraatkar et al (2022)	*35926992*	*excluded based on title relevance to the topic of interest*
Narouze and Souzdalnitski (2015)	*25650632*	*excluded based on title relevance to the topic of interest*
Benabbas et al (2017)	*28214369*	*non-cancer chronic pain epidemiology in specific body district,*
Vidale (2020)	*32852688*	*excluded based on title relevance to the topic of interest*
Rabbitts et al (2017)	*28363861*	*Records excluded after abstract reading*
Li et al (2021)	*34350974*	*non-cancer chronic pain epidemiology in specific body district,*
Al-Janabi et al (2021)	*34506696*	*excluded based on title relevance to the topic of interest*
Onesimo et al (2023)	*37758167*	*excluded based on title relevance to the topic of interest*
Fuglkjær et al (2017)	*29047337*	*non-cancer chronic pain epidemiology in specific body district,*
Abbate et al (2023)	*37094584*	*excluded based on title relevance to the topic of interest*
Stubbs et al (2015)	*25098864*	*excluded based on title relevance to the topic of interest*

Mohamed and Hairi (2015)	*25439125*	*Epidemiology of chronic pain in adults*
Eslick (2010)	*21111114*	
Evans et al (2019)	*31554017*	*excluded based on title relevance to the topic of interest*
Cazet et al (2018)	*29963937*	*Records excluded after abstract reading*
Stolzman et al (2015)	*25808780*	*excluded based on title relevance to the topic of interest*
Santos et al (2022)	*34791550*	*non-cancer chronic pain epidemiology in specific body district,*
Polick et al (2022)	*35779440*	*excluded based on title relevance to the topic of interest*
Kędra et al. (2021)	*32845380*	*non-cancer chronic pain epidemiology in specific body district,*
Pentapati et al (2021)	*32557184*	*Records excluded after abstract reading*
Andrews et al (2018)	*28815801*	*Epidemiology of chronic pain in adults*
French et al (2020)	*32362057*	*non-cancer chronic pain epidemiology in specific body district,*
Murray et al (2022)	*34817439*	*It is not possible to isolate the population of interest*
Barnish MS and Barnish J. (2016)	*26769789*	*excluded based on title relevance to the topic of interest*
Tsioutis(2017)	*27983969*	*excluded based on title relevance to the topic of interest*
Alsabri (2023)	*36775794*	*excluded based on title relevance to the topic of interest*

Isaacson (2023)	*33352607*	*excluded based on title relevance to the topic of interest*
Buldt AK and Menz H (2018)	*30065787*	*excluded based on title relevance to the topic of interest*
Oveisi (2018)	*37878813*	*Records excluded after abstract reading*
Sonneveld et al (2013)	*23412538*	*excluded based on title relevance to the topic of interest*
Vides-Rosales(2021)	*34749910*	*Records excluded after abstract reading*
Alsiri et al (2023)	*36462303*	*excluded based on title relevance to the topic of interest*
Bolsover et al (2014)	*23949010*	*excluded based on title relevance to the topic of interest*
Koxet al (2015)	*25872521*	*excluded based on title relevance to the topic of interest*
Strong M, and Johnstone P (2007)	*17636630*	*excluded based on title relevance to the topic of interest*
Lee et al (2016)	*26873725*	*Records excluded after abstract reading*
Olsen et al (2014)	*24297468*	*excluded based on title relevance to the topic of interest*
Soon et al (2013)	*23539047*	*non-cancer chronic pain epidemiology in specific body district,*
Cruz et al (2016)	*26223408*	*non-cancer chronic pain epidemiology in specific body district,*
Yeung et al (2015)	*26334643*	*excluded based on title relevance to the topic of interest*
Kordzadeh et al (2016)	*26951998*	*excluded based on title relevance to the topic of interest*

Dibello et al (2022)	*36579500*	*excluded based on title relevance to the topic of interest*
van der Slot et al (2021)	*32061920*	*Epidemiology of chronic pain in adults*
Mutiawati et al (2020)	*33953911*	*non-cancer chronic pain epidemiology in specific body district,*
Michaleff et al (2014)	*25070788*	*Records excluded after abstract reading*
Ng et al (2017)	*28028812*	*Records excluded after abstract reading*
Häggman-Henrikson et al (2013)	*23882454*	*non-cancer chronic pain epidemiology in specific body district,*
Peretz et al (2016)	*26611681*	*excluded based on title relevance to the topic of interest*
Bhatia et al (2014)	*24140926*	*excluded based on title relevance to the topic of interest*
Jackson et al (2015)	*26313056*	*Epidemiology of chronic pain in adults*
Mckinnon et al (2019)	*30508221*	*non-cancer chronic pain epidemiology in specific body district,*
Calvo-Muñoz et al (2012)	*23076081*	*non-cancer chronic pain epidemiology in specific body district,*
Burden et al (2016)	*26785915*	*excluded based on title relevance to the topic of interest*
Christidiset al (2019)	*30586192*	*Records excluded after abstract reading*

Louw et al (2017)	*27402957*	*Records excluded after abstract reading*
Rome et al (2010)	*20614443*	*excluded based on title relevance to the topic of interest*
Simpson and, Cook (2016)	*26626072*	*excluded based on title relevance to the topic of interest*
Vij et al (2022)	*35936798*	*excluded based on title relevance to the topic of interest*
Hincapié et al (2008)	*18760170*	*excluded based on title relevance to the topic of interest*
Young et al (2012)	*22727071*	*excluded based on title relevance to the topic of interest*
Jennings et al (2008)	*18937522*	*excluded based on title relevance to the topic of interest*
Jeffries et al (2007)	*17978666*	*Records excluded after abstract reading*
Lamichhane et al (2023)	*37811079*	*excluded based on title relevance to the topic of interest*
Graves and Gelband (2006)	*17054280*	*excluded based on title relevance to the topic of interest*
Banaschewski et al (2022)	*34635194*	*non-cancer chronic pain epidemiology in specific body district,*
Fishbain et al (2017)	*27188666*	*Epidemiology of chronic pain in adults*
Thakkar et al (2007)	*17222318*	*excluded based on title relevance to the topic of interest*

Roy et al (2007)	*28672087*	*excluded based on title relevance to the topic of interest*
Briggs et al (2009)	*19563667*	*Records excluded after abstract reading*
Li et al (2023)	*37747004*	*excluded based on title relevance to the topic of interest*
Hinze et al (2019)	*31606322*	*Records excluded after abstract reading*
Pacheco-Barrios(2019)	*36606574*	*Records excluded after abstract reading*
Beecham et al (2015)	*25768935*	*Epidemiology of chronic pain in adults*
Norton and Southon (2014)	*33148083*	*Records excluded after abstract reading*
Yamani and Olesen (2019)	*31307396*	*Records excluded after abstract reading*
Wilson et al (2004)	*15050886*	*excluded based on title relevance to the topic of interest*
Wong and Kinoshita (2006)	*16616342*	*excluded based on title relevance to the topic of interest*
Tsur et al (2024)	*37655587*	*excluded based on title relevance to the topic of interest*
Anand et al (2023)	*37659063*	*excluded based on title relevance to the topic of interest*
Pico et al (2023)	*37189914*	*Records excluded after abstract reading*
Onan and Ulger (2021)	*33570458*	*excluded based on title relevance to the topic of interest*
Joshee et al (2022)	*35086857*	*excluded based on title relevance to the topic of interest*
Ascough et al (2020)	*32201745*	*non-cancer chronic pain epidemiology in specific body district,*
Di Lorenzo et al (2005)	*15735476*	*Records excluded after abstract reading*

Gil and Tulandi (2020)	*31401265*	*Records excluded after abstract reading*
Abu-Arafeh et al (2010)	*20875042*	*Records excluded after abstract reading*
Gonnah etal (2023)	*37900548*	*excluded based on title relevance to the topic of interest*
Mantovani et al (2021)	*32555539*	*excluded based on title relevance to the topic of interest*
Chitkara et al (2005)	*16086724*	*Records excluded after abstract reading*
de Holanda et al (2023)	*36565536*	*excluded based on title relevance to the topic of interest*
Mao et al (2020)	*32405603*	*excluded based on title relevance to the topic of interest*
Venekamp et al (2018)	*29741289*	*excluded based on title relevance to the topic of interest*
Ploner et al (2020)	*32233273*	*Records excluded after abstract reading*
Abrahamsson (2013)	*24416880*	*excluded based on title relevance to the topic of interest*
Montardi C et al. (2024)	*38717582*	*Records excluded after abstract reading*
Robbertz AS et al. (2023)	*37515755*	*Records excluded after abstract reading*
Mohammadi P et al. (2023)	*37106419*	*Epidemiology of chronic pain in adults*
Faingold R et al. (2004)	*15663319*	*Epidemiology of chronic pain in adults*
Kaplan SA et al. (2013)	*23409689*	*Records excluded after abstract reading*
Albers et al,.	*25002234*	*excluded based on title relevance to the topic of interest*
Sim et al,. (2024)	*38738779*	*excluded based on title relevance to the topic of interest*
Velasco et al,. (2023)	*35961509*	*excluded based on title relevance to the topic of interest*
Simon et al,. (2013)	*23742735*	*excluded based on title relevance to the topic of interest*

Loring et al., (2020)	*33371949*	*excluded based on title relevance to the topic of interest*
Dagenais et al,. (2008)	*19037710*	*Records excluded after abstract reading*
Ahrari et al,. (2021)	*34816280*	*excluded based on title relevance to the topic of interest*
Armbrust W et al (2016)	*26656031*	*excluded based on title relevance to the topic of interest*
Kariyanna et al (2021)	*32910756*	*excluded based on title relevance to the topic of interest*
Gatti S et al (2022)	*35267970*	*excluded based on title relevance to the topic of interest*
Reinfjell T et al (2014)	*31423647*	*non-cancer chronic pain epidemiology in specific body district,*
Dépelteau et al,. (2020)	*31706663*	*excluded based on title relevance to the topic of interest*
Jauregui et al,. (2020)	*33094401*	*excluded based on title relevance to the topic of interest*
Walsh et al,. (2015)	*25867816*	*excluded based on title relevance to the topic of interest*
Lim et al,. (2022)	*36135926*	*excluded based on title relevance to the topic of interest*
Steele et al,.(2006)	*16418645*	*excluded based on title relevance to the topic of interest*
Walton et al,. (2021)	*33593705*	*excluded based on title relevance to the topic of interest*
Koljonen et al,. (2009)	*18094970*	*excluded based on title relevance to the topic of interest*
Liu et al,. (2009)	*19285858*	*excluded based on title relevance to the topic of interest*
Roy et al (2023)	*37539955*	*excluded based on title relevance to the topic of interest*
Petzold and plant (2014)	*23700317*	*excluded based on title relevance to the topic of interest*

Chabbert et al,. (2021)	*32475156*	*excluded based on title relevance to the topic of interest*
Campos et al,. (2022)	*36554016*	*excluded based on title relevance to the topic of interest*
MacLean et al,. (2013)	*23273876*	*excluded based on title relevance to the topic of interest*
Pigozzi et al,. (2021)	*33691709*	*excluded based on title relevance to the topic of interest*
Ferguson et al,. (2019)	*31727033*	*excluded based on title relevance to the topic of interest*
Mastoraki et al,. (2021)	*34137134*	*excluded based on title relevance to the topic of interest*
Arthur et al,. (2018)	*30416015*	*excluded based on title relevance to the topic of interest*
Beveridge et al,. (2024)	*38112571*	*excluded based on title relevance to the topic of interest*
Sanchis-Alfonso et al,. (2022)	*35429242*	*excluded based on title relevance to the topic of interest*
Díaz-Mohedo E et al,. (2011)	*21917358*	*excluded based on title relevance to the topic of interest*
Kamphorst et al,. (2021)	*33748435*	*excluded based on title relevance to the topic of interest*
Paixão et al,. (2018)	*30007303*	*excluded based on title relevance to the topic of interest*
Huckerby L et al (2023)	*36968631*	*excluded based on title relevance to the topic of interest*
Martins et al,. (2020)	*31397485*	*excluded based on title relevance to the topic of interest*
Fair et al,. (2019)	*31807093*	*excluded based on title relevance to the topic of interest*
Navarro-Pérez et al,. (2020)	*32672349*	*excluded based on title relevance to the topic of interest*

Behnood et al., (2022)	*34813820*	*excluded based on title relevance to the topic of interest*
Hauskov Graungaard et al., (2019)	*30690768*	*excluded based on title relevance to the topic of interest*
Harbottle et al., (2022)	*36645779*	*excluded based on title relevance to the topic of interest*
Calvo-Muñoz I et al (2018)	*28915154*	*excluded based on title relevance to the topic of interest*
Simms et al., (2011)	*21620647*	*excluded based on title relevance to the topic of interest*
Oosterhoff et al (2022)	*32603198*	*excluded based on title relevance to the topic of interest*
Smid et al., (2014)	*25442977*	*excluded based on title relevance to the topic of interest*
Zuvela et al., (2018)	*29468835*	*excluded based on title relevance to the topic of interest*
Minervini et al., (2023)	*37732431*	*excluded based on title relevance to the topic of interest*
Novak et al (2012)	*23045562*	*excluded based on title relevance to the topic of interest*
Villablanca P et al (2015)	*26730299*	*excluded based on title relevance to the topic of interest*
Van den Heuvel et al,. (2020)	*32355138*	*excluded based on title relevance to the topic of interest*
Sanchez-Espino et al., (2024)	*37750181*	*excluded based on title relevance to the topic of interest*
Singh et al., (2022)	*34268659*	*excluded based on title relevance to the topic of interest*
Kitschen et al., (2024)	*38268231*	*excluded based on title relevance to the topic of interest*
Kaufman et al., (2016)	*26938639*	*excluded based on title relevance to the topic of interest*

Lionetti et al., (2010)	*20345955*	*excluded based on title relevance to the topic of interest*
Brink et al., (2022)	*34157947*	*excluded based on title relevance to the topic of interest*
Omara et al., (2021)	*32757443*	*excluded based on title relevance to the topic of interest*
Song et al., (2018)	*30125394*	*excluded based on title relevance to the topic of interest*
Orsoet al., (2022)	*35213607*	*excluded based on title relevance to the topic of interest*
Li et al., (2023)	*36735342*	*excluded based on title relevance to the topic of interest*
Jonassaint et al., (2016)	*26991317*	*excluded based on title relevance to the topic of interest*
Thambapillary et al., (2013)	*23523494*	*excluded based on title relevance to the topic of interest*
Jung et al., (2010)	*20697189*	*excluded based on title relevance to the topic of interest*
Harbottle et al., (2022)	*36645779*	*excluded based on title relevance to the topic of interest*
Moudgalya et al,.(2024)	*39372532*	*excluded based on title relevance to the topic of interest*
Alzahrani et al. (2019)	*31160632*	*excluded based on title relevance to the topic of interest*
Jacob et al. (2025)	*39095515*	*excluded based on title relevance to the topic of interest*
Armour et al. (2019)	*31339951*	*excluded based on title relevance to the topic of interest*
Wilk et al. (2024)	*39469820*	*excluded based on title relevance to the topic of interest*
Waugh et al. (2013)	*24286461*	*excluded based on title relevance to the topic of interest*

Storebø et al. (2018)	*29744873*	*excluded based on title relevance to the topic of interest*
Davis et al. (2005)	*16093745*	*excluded based on title relevance to the topic of interest*
Calvo-Muñoz et al,. (2020)	*31421006*	
Carter et al. (2020)	*32887663*	*excluded based on title relevance to the topic of interest*
Shaari et al,. (2024)	*39049518*	*excluded based on title relevance to the topic of interest*
Jälevik et al,. (2022)	*34110616*	*excluded based on title relevance to the topic of interest*
Gaziev et al,. (2013)	*24274173*	*excluded based on title relevance to the topic of interest*
van Meegeren et al,. (2014)	*24533951*	*excluded based on title relevance to the topic of interest*
Li et al,. (2017)	*27928615*	*excluded based on title relevance to the topic of interest*
Canton et al,. (2021)	*34313663*	*excluded based on title relevance to the topic of interest*
Abebeet al,. (2024)	*39567941*	*excluded based on title relevance to the topic of interest*
Firman et al,. (2022)	*36053659*	*excluded based on title relevance to the topic of interest*
Bailey & McManus (2008)	*18496120*	*excluded based on title relevance to the topic of interest*
Pouraliroudbaneh et al,. (2024)	*38654586*	*excluded based on title relevance to the topic of interest*
Alsabri et al,. (2024)	*38626180*	*excluded based on title relevance to the topic of interest*
Evensen et al,. (2022)	*35867340*	*excluded based on title relevance to the topic of interest*
Wimalasiri-Yapa et al,. (2020)	*33036370*	*excluded based on title relevance to the topic of interest*

Peymani et al,. (2019)	*30102073*	*excluded based on title relevance to the topic of interest*
Tam et al,. (2020).	*33010713*	*excluded based on title relevance to the topic of interest*
Zis et al,. (2018)	*30301194*	*excluded based on title relevance to the topic of interest*
Anyachukwu et al,. (2024)	*38308207*	*excluded based on title relevance to the topic of interest*
Liang et al,. (2024)	*38183517*	*excluded based on title relevance to the topic of interest*
Majava et al,. (2024)	*39320539*	*excluded based on title relevance to the topic of interest*
Alrashd et al,. (2024)	*38483754*	*excluded based on title relevance to the topic of interest*
Saatci et al,. (2024)	*38862882*	*excluded based on title relevance to the topic of interest*
Wang and Yuan (2021)	*33725961*	*excluded based on title relevance to the topic of interest*
Bredow et al,. (2016)	*27075679*	*excluded based on title relevance to the topic of interest*
Luo et al,. (2022)	*35909298*	*excluded based on title relevance to the topic of interest*
Yamaki et al,. (2020)	*32206904*	*excluded based on title relevance to the topic of interest*
Aşci et al,. (2024)	*38556938*	*excluded based on title relevance to the topic of interest*
Vanwesemael et al,. (2023)	*37947469*	*excluded based on title relevance to the topic of interest*
Hamed et al,. (2024)	*39184654*	*excluded based on title relevance to the topic of interest*
Ronsivalle et al,. (2024)	*38012098*	*excluded based on title relevance to the topic of interest*

Wasserstein et al,. (2015)	*26131297*	*excluded based on title relevance to the topic of interest*
Leboeuf-Yde and Lauritsen (1995)	*8588168*	*excluded based on title relevance to the topic of interest*
Amaral et al,. (2021)	*33170020*	*excluded based on title relevance to the topic of interest*
Barik et al,. (2023)	*36947313*	*excluded based on title relevance to the topic of interest*
Graham et al,. (2018)	*30522126*	*excluded based on title relevance to the topic of interest*
Kalach et al,. (2021)	*34338237*	*excluded based on title relevance to the topic of interest*
Patterson et al,. (2018)	*30143272*	*excluded based on title relevance to the topic of interest*
Cabana et al,.(2021)	*34874412*	*excluded based on title relevance to the topic of interest*
Tassi et al,. (2020)	*31914331*	*excluded based on title relevance to the topic of interest*
Alexiou et al,. (2022)	*35829838*	*excluded based on title relevance to the topic of interest*
Kacmaz and Kaçmaz (2024)	*36786143*	*excluded based on title relevance to the topic of interest*
Dowling et al,. (2009)	*19187397*	*excluded based on title relevance to the topic of interest*
